# Synergistic Effect and Antiquorum Sensing Activity of *Nymphaea tetragona* (Water Lily) Extract

**DOI:** 10.1155/2014/562173

**Published:** 2014-05-08

**Authors:** Md. Akil Hossain, Ji-Yong Park, Jin-Yoon Kim, Joo-Won Suh, Seung-Chun Park

**Affiliations:** ^1^Laboratory of Clinical Pharmacokinetics and Pharmacodynamics, Department of Pharmacology, College of Veterinary Medicine, Kyungpook National University, Daegu 702-701, Republic of Korea; ^2^Institute of Clean Bio, Daejeon 301-212, Republic of Korea; ^3^Division of Bioscience and Bioinformatics, Myongji University, Science Campus, Gyeonggi 449-728, Republic of Korea

## Abstract

Salmonellosis is a common and widely distributed food borne disease where *Salmonella typhimurium* is one of the most important etiologic agents. The purpose of this study was to investigate the antimicrobial activity of *Nymphaea tetragona* alone and in combination with antibiotics against *S. typhimurium*. It also aimed to assess the plant for quorum sensing inhibition (QSI) activity and to identify the bioactive compounds. The antibacterial activities of the extract were assessed using broth microdilution method. Disk agar diffusion method was employed to determine the QSI and bioactive compounds were identified by GC-MS analysis. Ethyl acetate fraction of *N. tetragona* extract (EFNTE) demonstrated good antimicrobial activity (MIC 781 **μ**g/mL) against 4 strains out of 5. FIC index ranged from 0.375 to 1.031 between EFNTE/tylosin and 0.515 to 1.250 between EFNTE/streptomycin against *S. typhimurium*. Among all extracts, EFNTE and butanol fraction more significantly inhibited pigment production of *C. violaceum*. Polyphenols were identified as major compound of EFNTE and butanol fraction. These results indicate that combination among *N. tetragona* extract and antibiotics could be useful to combat drug-resistance *Salmonella* infections and polyphenols are promising new components from *N. tetragona* that warrant further investigation as a candidate anti-*Salmonella* agent and quorum sensing inhibitor.

## 1. Introduction

*Salmonella* species are the leading cause of bacterial gastroenteritis in humans and animals all over the world [1, 2]. Food animals and water are the most important reservoirs of the bacteria [2] where the outbreaks of* Salmonella* infections have increasingly been associated with processed foods [3, 4]. There are 1300 million cases of gastroenteritis, 16 million cases of typhoid fever, and 3 million cases of deaths worldwide each year due to* Salmonella* infections [5].* Salmonella typhimurium* is one of the most common serovars associated with clinically reported salmonellosis in humans in most parts of the world, accounting for at least 15% of infections [2, 6].

*S. typhimurium* infects a wide range of animal hosts, including poultry, cattle, and pigs, and is termed ubiquitous which usually causes a self-limited gastroenteritis in humans [7]. The use of antibiotics is a major strategy and they are commonly used therapeutically and prophylactically to treat* S. typhimurium* infections in human and animal. However, increased antimicrobial resistance is exacerbating impact on public health worldwide, which leads to increased morbidity, mortality, and treatment costs [8, 9]. Scientific studies showed that tylosin has low or no inhibitory effects on experimentally inoculated* S. typhimurium* in pigs [10, 11]. It was also reported that* S. typhimurium* illustrated resistance to ampicillin, chloramphenicol, streptomycin, sulphonamides, and tetracycline in 1980s, in the UK and later distributed extensively through Europe and North America. Additional resistance had attained to trimethoprim-sulfamethoxazole, ciprofloxacin, and extended-spectrum cephalosporins within the 1990s [12]. Development of alternative antibacterial therapies is necessary to overcome this outbreak. Approximately 80% of the world's inhabitants rely on traditional medicine for their primary health care and plants also play an important role in the health care system [13]. The possible therapeutic use of* Nymphaeaceae* may be a good alternative of traditional antibacterial.

The Nymphaeaceae also called water lilies have a broad range of flower colors and are living on the banks of lakes and rivers, distributed in tropical areas around the world [14–16]. A number of species of* Nymphaea* in Nepal, India, and China are thought to act as functional drug plants [17]. Many bioactive and pharmacologically important compounds have been obtained from* Nymphaea* species and used in medicine and pharmacy [18]. Flower extract of* N. nouchali *which possess compounds with high antibacterial and cytotoxic properties [19, 20] and ethyl acetate extract of* N. nouchali* leaf extract have antibacterial activity against a wide range of strains [21].* Nymphaea lotus* extract was reported to have bioactive compounds such as tannins, flavonoids, alkaloids, anthraquinones, saponins, cardiac glycosides, and phenolics where methicillin and vancomycin resistant* S. aureus*,* S. pyogenes,* and* E. coli* were highly susceptible to* N. lotus* [13, 22, 23]. The pygmy water lily,* Nymphaea tetragona* (Ait.) Georgi (Nymphaceae), is one of the widely distributed plants, ranging globally from Asia-temperate, Asia-tropical, Europe, and northern America [24]. It has ethnomedical uses as the rhizome is used to cure acute diarrhea and dysentery by tribal herbal practitioners in Indian region [24].

Although there are many reports in the ethnomedicinal values of* Nymphaea*, information on their antibacterial efficacy is scarce and with low scientific caliber for further commercial use. Hence, it is important to determine the antibacterial activity very clearly and to identify the antibacterial active compounds of this plant. Furthermore, the potentials of water lily in combating antimicrobial resistance alone and in combination with antibiotics and the inhibition of quorum sensing controlled virulent factors of microbial pathogens were not explored previously. Thus, the current study was designated to evaluate the antibacterial activity of* Nymphaea tetragona* extract alone and in combination with commercial antibiotics against* Salmonella typhimurium. *Quorum sensing inhibition activity of the extract against biomonitor strain* Chromobacterium violaceum* was also aimed at assessing in this study. Finally, a GC-MS analysis was performed to identify and quantify the major compounds of the* N. tetragona*.

## 2. Materials and Methods

### 2.1. Bacterial Strains and Culture Medium

*Salmonella typhimurium* QC strain KTCC2515 and clinical isolates ST171, ST482, ST688, and ST21A were used for experiments in this study (Table 1) which were collected from different farms in Republic of Korea. Bacterial strains were suspended in Mueller Hinton broth (MHB, Difco, USA) and then incubated at 37°C with 200 rpm for 20 h. Mueller Hinton agar (MHA, Difco) was used for the agar diffusion method.

### 2.2. Plant Extraction and Fractionation

*N. tetragona* powder of body and root mixture was purchased from Chamsamgol Lotus Farm (Chungju, Republic of Korea). 100 g of the powdered material was boiled with 1000 mL of 50% methanol in a 2000 mL three-neck round bottom boiling flask (Schott Duran, NY, USA) at 100°C setting temperature on nonasbestos surface for 3 h when the % Brix and absorbance of the extract became the highest. The supernatant of* N. tetragona* 50% methanol extract (NTME) was collected by filtration (70 mm, Advantec; Toyo Roshi Kaisha Ltd., Tokyo, Japan) and solid particles retained on the filter were discarded. The solvent was then removed under reduced pressure in Buchi Rotavapor R-114 (BUCHI Labortechnik AG in Flawil, Switzerland) at 10 rpm and Eyela CCA-1111 (Tokyo Rikakikai Co. Ltd., made in China) and solidified by freeze-drying prior to use. The yield of extract was 10.71%.

10 g of lyophilized* N. tetragona *extract was suspended in 50 mL of water and fractionated with equivalent amount of dichloromethane, ethyl acetate, and butanol correspondingly by separating funnel. The solvent fractions were solidified and the distribution of extract was 0.43% in dichloromethane, 4.01% in ethyl acetate, and 46.74% in butanol. 47.36% was obtained as residue in water and 1.46% was process loss.

### 2.3. Antibacterial Resistance Testing

The disk-agar diffusion method validated by the Clinical and Laboratory Standards Institute [25] was used to verify the resistance pattern of four* S. typhimurium* clinical isolates against different commercial antibiotics.* S. typhimurium* KTCC 2515 was used as quality control strain in this experiment. Antibiotic sensitivity was considered according to the zone diameter interpretative standards of CLSI [26].

### 2.4. Determination of MIC and MBC

MIC was determined by the standard broth microdilution method according to the CLSI guidelines [27] in MHB using ~5 × 10^5^ CFU/mL of inoculums concentration. The MHB was supplemented with serial dilutions ranging from 24.4–25000, 4.9–2500, 12.2–6250, and 4.9–25000 *μ*g/mL, respectively, for NTME, DFNTE, EFNTE, and BFNTE in 100 *μ*L volumes in 96-well plates. Bacterial suspensions were adjusted to 0.1 OD at 600 nm, diluted 1/100, and dispensed in 100 *μ*L aliquots to all the wells, including drug-free controls. Initial CFU/mL of the bacterial suspensions was determined by plating 10-fold dilutions on MHA plates. After overnight incubation at 37°C, inoculums from each well were diluted 10-fold and plated on MHA plates to determine the CFU/mL of each well. MIC was determined by comparing the final CFU with initial CFU. The lowest concentration of the extract inhibiting the increase of CFU was considered as MIC. 100 *μ*L drug dilutions from wells of 96-well plates were cultured on MHA plates to determine the existing bacterial number. MBC was considered as the lowest concentration which can eradicate 99.9% bacteria [28].

### 2.5. Killing Rate of* Salmonella typhimurium*

Killing rate was evaluated as previously described by Tia Dubuisson et al., [28] with some modification. 0.25 × MIC, 0.5 × MIC, 1 × MIC, and 2 × MIC of EFNTE were prepared in 10 mL of MHB. Four-hour old* S. typhimurium* (KTCC 2515) cultures were adjusted to 0.1 OD in 600 nm, diluted to make suitable concentration which will be 10^6^ CFU/mL after inoculating to each tube. Incubation of tubes was done at 200 rpm on a shaker incubator at 37°C. In 0, 1, 2, 3, 4, 6, 8, 12, and 24 hours, the CFU of the cultures was determined by culturing 20 *μ*L aliquots of 10-fold dilutions on MHA plates. Plates were incubated at 37°C overnight before counting CFU. The mean log_10_ CFU/mL for each extract was plotted against different times.

### 2.6. *Nymphaea tetragona* Extract and Antimicrobial Combination Activities* In Vitro*

Combination effects of EFNTE with ampicillin, marbofloxacin, norfloxacin, Streptomycin, trimethoprim, and tylosin (Sigma-Aldrich Co. St. Louis, MO, USA) against* S. typhimurium* were screened out, since these antimicrobials have reported for becoming resistant to* S. typhimurium* [10–12]. Previously described double disk agar diffusion method [29] was used with slight modification. A zone diameter of individual antimicrobial was determined by CLSI guideline [25].

Disks were placed separately with a distance equal to the sum of the zone radii for each disk tested separately. The interface of inhibition zones was observed after incubation. Generally, synergism shows an improved zone, indifference shows no change, and antagonism shows a reduced zone compared to the zones of individual test [29]. Further test of synergism was done only for tylosin and streptomycin with the extract fraction, as those 2 antibiotics showed synergism in disk diffusion test.

Combination interactions of the extract fraction with tylosin and streptomycin were determined in 96-well plates by previously described chequerboard microdilution method with slight modifications [30]. Antibiotic was vertically and the extract was horizontally diluted to get a matrix of different combinations of 2 antibacterials. Similar dilutions of individual drugs and the drug-free medium control were included in each test plate. Plates were incubated at 35°C for 16 to 20 hours after the addition of ~5 × 10^5^ CFU/mL of inoculums. From the MIC of the drugs alone and in combination, we calculated the fractional inhibitory concentration (FIC) and the FIC index (FICI). FIC is the MIC in combination divided by the MIC of the individual drug and FICI is the sum of the FICs of individual drugs. An FICI of ≤0.5 is considered synergistic, an FICI of 4.0 is considered antagonistic, and an FICI of 0.5–4 is considered to indicate no interaction [31].

### 2.7. Quorum Sensing Inhibition

Quorum sensing inhibition of NTME and its solvent fractions were verified in accordance with the method described by Alvarez et al. [32].* C. violaceum* CV12472 was employed to find out the pigment inhibition of extracts for attaining a qualitative screening. 100 *μ*L of fresh culture diluted as 2.5 × 10^6^ CFU/mL was poured on media for the preparation of LB agar plates. 60 *μ*L of each extract with specific concentrations was applied to saturate the sterile paper disks (8 mm). Normal saline (60 *μ*L) was used as negative control and purified furanone (100 *μ*g) was applied as positive control whereas tetracycline (10 *μ*g) was employed to compare antibacterial and antiquorum sensing activity. Inhibition of pigment production around the disc was checked after 18–24 h incubation at 30°C. The sensitivity to different agents was classified by the diameter of the inhibition zones as follows: “not sensitive” for diameter less than 8 mm, “sensitive” for diameter between 9 and 14 mm, “very sensitive” for diameter between 15 and 19 mm, and “extremely sensitive” for diameter larger than 20 mm [33, 34].

### 2.8. Gas Liquid Chromatography Coupled Mass Spectrophotometric (GC-MS) Analysis

GC-MS analysis of DFNTE, EFNTE, and BFNTE was performed by “Center for Scientific Instruments” of Kyungpook National University and carried out using a HP 6890 Plus GC gas chromatograph with a (MSD)—HP 5973 MSD mass selective detector (Hewlett-Packard). Samples were diluted 1 : 1000 (v : v) with HPLC grade dichloromethane. Aliquots of the sample (1 *μ*L) were injected into an HP-5 column. The GC oven temperature was set at 50°C for 4 min, increased to 280°C at a rate of 4°C/min, and held at the final temperature for 2 min. Velocity of the He carrier gas (99.99%) was 0.7 mL/min. Quantitative analysis was performed using the area normalization method.

### 2.9. Statistical Analysis

The mean values and the standard deviation were calculated from the data obtained from triplicate trials. Analysis of variance (ANOVA) was used to verify differences and* F*-test was applied for the determination of statistical significance between groups.

## 3. Results

### 3.1. MIC and MBC of* Nymphaea tetragona* 50% Methanol Extract

The antimicrobial activity of NTME and its solvent fractions were confirmed by determining the minimum inhibitory concentration (MIC) and minimum bactericidal concentration (MBC) against different strains of* S. typhimurium*. The results indicated that the EFNTE possessed the strongest antibacterial activity among all fractions. MBC of EFNTE against all tested* S. typhimurium* strains was ≤1562 *μ*g/mL, whereas the MIC values were within 781 *μ*g/mL for all strains except one clinical isolate (Table 2).

### 3.2. Killing Rate of* Salmonella typhimurium*

**Figure 1:**
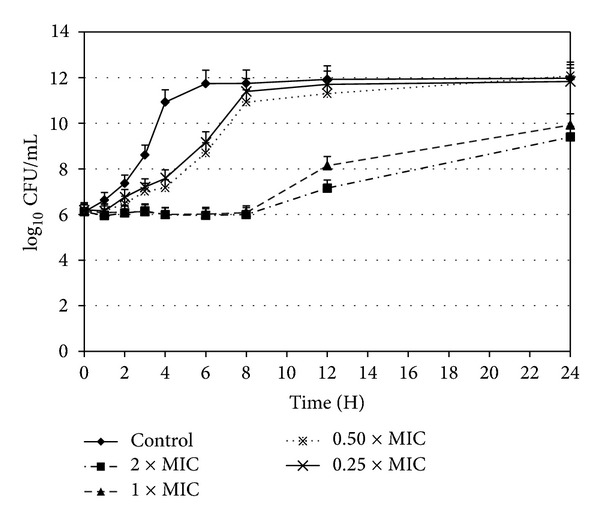
Time-kill curves of* S. typhimurium* KCTC 2515 after treatment with EFNTE at 0.25 × MIC (*※*), 0.50 × MIC (×), 1 × MIC (▲), and 2 × MIC (■). Ca-MHB was used as a control (◆). The results are presented as mean ± standard deviations (*n* = 3), and the coefficients of variation for all concentrations are ≤5%.

Time-kill curves of* S. typhimurium* after treatment with EFNTE are demonstrated in Figure 1. The EFNTE of both the 1 × MIC and 2 × MIC concentrations showed complete inhibition up to 8 hours and started their log phase from this time point. At 24 hours, the growth level was 2-fold lower in 1 × MIC and 3-fold lower in 2 × MIC of EFNTE than the growth control. At 0.25 × MIC and 0.5 × MIC, bacteria reached log phase after 1 hour and stationary phase after 8 hours whereas the control started stationary phase after the 4th hour. None of the tested concentrations showed complete killing effect within 24 h.

### 3.3. *In Vitro* Synergy with Commercial Antimicrobials

**Figure 2:**
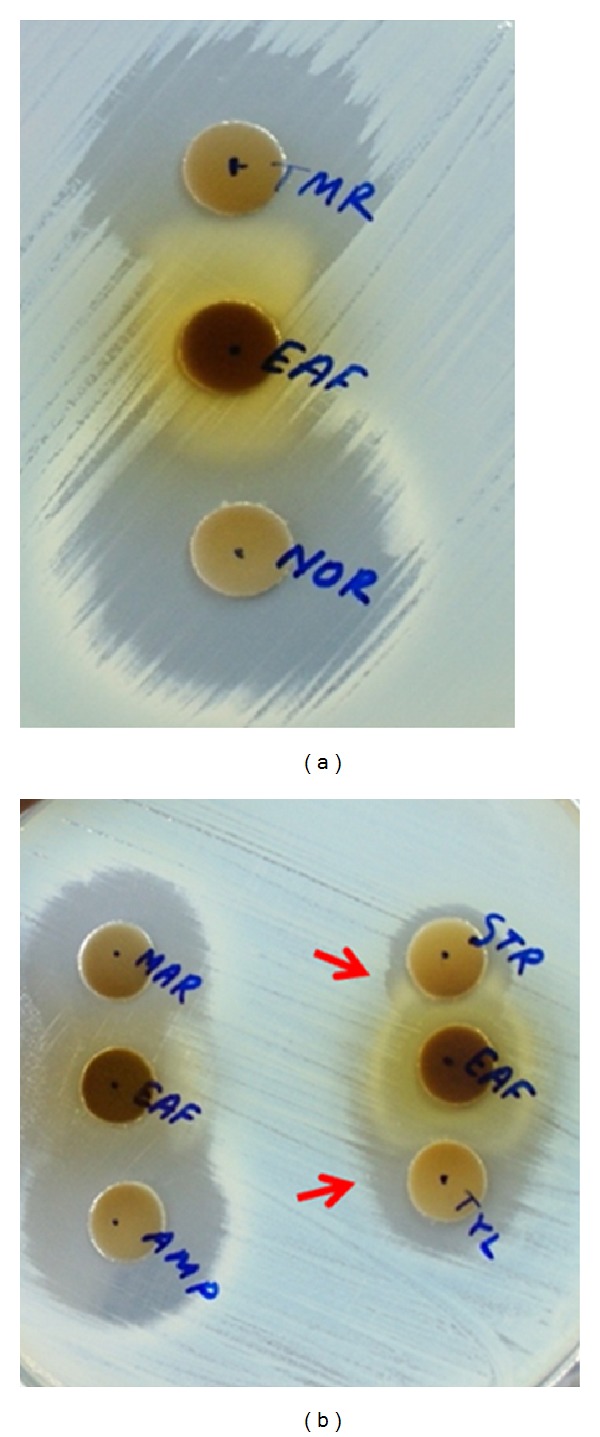
Double disk synergy of EFNTE with commercial antibiotics against* S. typhimurium* KCTC 2515. EAF: ethyl acetate fraction of* N. tetragona* 50% methanol extract; TMR: trimethoprim; NOR: norfloxacin; MAR: marbofloxacin; AMP: ampicillin; STR: streptomycin; and TYL: tylosin. Red arrows indicate synergistic interactions.

To investigate whether there is any synergy between EFNTE and commercial antibiotics, we examined six antibiotics with EFNTE by disc diffusion method. EFNTE exhibited synergism only with tylosin and streptomycin and has additive/indifferent effects with other antibiotics. There was no antagonistic effect observed in EFNTE with those antibiotics (Figure 2).

**Figure 3:**
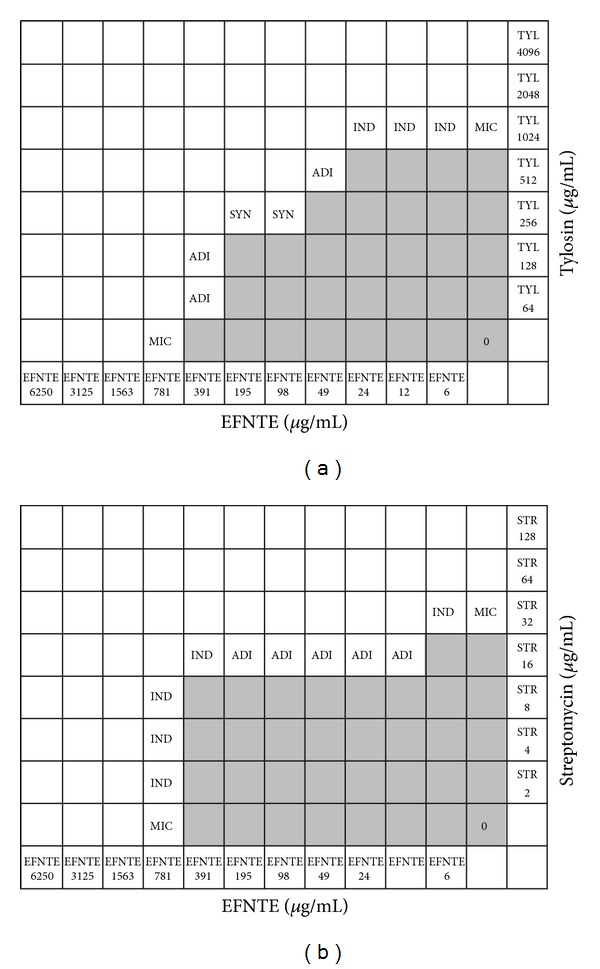
Combination interaction of EFNTE with (a) tylosin and (b) streptomycin against* S. typhimurium* KCTC 2515 by checkerboard microdilution method. Ash colors indicate bacterial growth and without color zones are free of bacteria. EFNTE: ethyl acetate fraction of* N. tetragona* 50% methanol extract; TYL: tylosin; STR: streptomycin; SYN: synergistic effect; ADI: additive effect; IND: indifferent effect; MIC: minimum inhibitory concentration.

To reconfirm the synergistic activity of EFNTE with tylosin and streptomycin, we further performed checkerboard microdilution assay. The results of the checkerboard analysis are summarized in Tables 3 and 4 and Figure 3. An increased sensitivity against tylosin was observed in combination with EFNTE. The corresponding FICIs were ≤0.5 in tested strain, demonstrating a synergistic effect. However, EFNTE in combination with streptomycin showed additive and indifferent instead of synergistic interaction.

### 3.4. Quorum Sensing Inhibition of NTME

The inhibition diameter of* C. violaceum* pigment in presence of NTME and solvent fractions of NTME is presented in Table 5.* C. violaceum* showed sensitivity to all fractions including the crude extract. Although the crude extract and all fractions of extract significantly prevented the pigment, ethyl acetate and butanol fractions showed highest inhibition among all. Tetracycline demonstrated bactericidal, furanone confirmed pigment inhibitory effect, and the negative control had no activity.

### 3.5. Gas Liquid Chromatography Coupled Mass Spectrophotometric (GC-MS) Analysis

**Figure 4:**
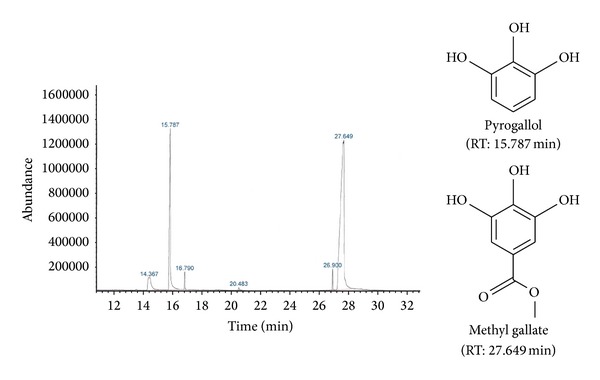
Gas chromatography coupled with mass spectroscopy (GC-MS) chromatogram of EFNTE and chemical structure of major compounds.

The major identified compounds with their biological activities are illustrated in Table 6 according to their elusion order. The major chemical compounds in DFNTE were mainly hydrocarbons (about 46.46%) and EFNTE contains methyl gallate (70.44%), 1, 2, 3-benzenetriol or pyrogallol (20.61%), and 6, 8-dimethylbenzocyclooctene (5.90%). The major compounds found in BFNTE were 2-hydrazinoquinoline (57.61%), pyrogallol (20.09%), and methyl gallate (12.77%). GC-MS chromatogram of EFNTE and chemical structures of methyl gallate and pyrogallol are presented in Figure 4. We have shown only the chromatogram of EFNTE and structures of those compounds as they are expected for desired effects and are abundant in EFNTE.

## 4. Discussion

There are recent reports indicating the resistance of several bacterial strains against different antibiotics that have been used in the treatment of infectious diseases of human and animals [45]. Thus, to combat infectious diseases associated with resistant pathogens, development of alternative antimicrobial drugs is urgent [46, 47]. The* in vitro* activity of EFNTE against resistance strains of* Salmonella typhimurium* (Table 1) reflects that the plant could be a good candidate as a source of active phytoconstituents to minimize the development of bacterial resistance and to ensure clinical cure of bacterial infection.

In the present study, MIC results of EFNTE exhibited antibacterial activity against all strains of* S. typhimurium* tested which have been shown to be resistant in one to six out of eight antibiotics (Table 1). The time-kill assay also exposed that the extract fraction effectively inhibited the growth of* S. typhimurium*. Utilities of EFNTE are again explored by the combination interactions with commercial antibiotics where it possessed synergistic effect with tylosin and additive and/or indifferent effects with other tested antibiotics. Quorum sensing inhibition activity of NTME and solvent fractions of the extract demonstrated varied level of effects statistically wherever EFNTE and BFNTE showed the greatest activity among all fractions and crude extract. Furthermore, the GC-MS analysis was performed to investigate possible components from the NTME for its antibacterial potential. This GC-MS analysis efficiently confirmed the existence of some major phenolic compounds (methyl gallate and pyrogallol) along with several other minor constituents.

MIC and MBC of NTME and its solvent fractions were studied against 5 strains of* S. typhimurium*. The results in Table 2 indicated that the EFNTE possessed the strongest antibacterial activity among all the fractions and crude extract. Considering the antibacterial activity, EFNTE was selected for further investigation. Extract having MIC values less than 8 mg/mL was believed as active crude extract [48]. Again, it was suggested to avoid the MIC greater than 1 mg/mL for crude extract and 0.1 mg/mL for isolated compounds. 0.1 mg/mL and 0.01 mg/mL MICs correspondingly for crude extract and isolated compounds would be very interesting activity [49]. Including all, MIC values less than 1 mg/mL were considered as good activity in the current study.

Time-kill curves have been used to determine whether the effects of EFNTE are either bacteriostatic or bactericidal and are useful for the evaluation of the pharmacodynamic characteristics of new antimicrobial agents [50]. According to our results, EFNTE shows bacteriostatic activity against the tested bacteria, since a reduction ≥99.9% of the inoculums was not observed compared to the growth control (Figure 1). At its 1 × MIC and 2 × MIC, EFNTE was able to inhibit* S. typhimurium* in the first 8 h of incubation. Then, 1 × MIC and 2 × MIC of EFNTE treated groups started their log phase. At the end of the incubation period (24 h), 3-fold reduced inoculums concentration in 2 × MIC of EFNTE and 2-fold lower inoculums concentration in 1 × MIC of EFNTE were achieved in contrast to control, indicating that EFNTE displays a bacteriostatic effect.

It is always suggested to treat bacterial infections with a combination of antimicrobial agents for the prevention of drug resistance development and to improve efficacy. Drug combinations having synergistic interactions are generally considered as more effective and, therefore, preferable [28]. Incidentally, EFNTE is proved to have synergistic effects with tylosin against* S. typhimurium*. The EFNTE also has additive and indifferent effects with streptomycin and other antibiotics tested against the same bacterial strain and has no antagonistic interactions with those antibiotics. Excellent* in vitro* activity combined with synergistic effects with other antibacterial drugs underscores the potential utility of EFNTE for the treatment of* Salmonella* associated infections.

In this study, NTME and its solvent fractions also possessed quorum sensing inhibition activity. EFNTE and BFNTE are considered as very sensitive or sensitive among them, whereas the crude extract and the rest of the fractions are sensitive according to the previous report [33]. Every fraction of extract may have many major and minor compounds with QS-inhibition activity which is reflected in the result. Furthermore, many compounds having antimicrobial activity were identified by the GC-MS analysis of DFNTE, EFNTE, and BFNTE. Methyl gallate, pyrogallol, and some hydrocarbons (hexacosan, heptacosan, octacosan, etc.) are important in response to desired type of effects and the proportion of those compounds in extracts. Pyrogallol is reported to have quorum sensing inhibition activity [36, 37] and antimicrobial activity [38] that is present in both EFNTE (20.61%) and BFNTE (22.09%). Methyl gallate is identified from EFNTE (70.44%) and BFNTE (12.77%) which is an antibacterial agent [43]. The scenario is now clear that methyl gallate and pyrogallol were contributing the vital role of the activity of EFNTE where they occupy about 91% of the total. The similar level of QS inhibition activity of EFNTE and BFNTE may be because of the presence of pyrogallol almost in equivalent amount in both fractions. The combined activity of those hydrocarbons or minor compounds present in DFNTE may possess the anti-QS activity.

This is the first study describing the combination interaction with commercial antibiotics and quorum sensing inhibition activity of* N. tetragona* extract. This study is also reporting the existence of methyl gallate and pyrogallol for the first time from this species. Together with all the promising* in vitro* assay findings, we believe that* N. tetragona* 50% methanol extract is expected to become a novel antimicrobial treatment for* Salmonella* infection of animals and human. Further study is needed to explore the mechanism of quorum sensing inhibition, antibacterial, and synergistic activity of the extract.* In vivo* activity and cytotoxic effects of the extract are also necessary to expose.

## Figures and Tables

**Table 1:** List of *Salmonella *strains used in this study along with their sources and sensitivities against commercial antibiotics.

Antimicrobials	Concentration (*μ*g/disk)	KCTC2515	Isolates			
			ST171 (chicken)	ST482 (pig)	ST688 (cattle)	ST21A (pig)
Chloramphenicol	30	S	S	S	R	S
Ciprofloxacin	5	S	S	S	S	S
Erythromycin	15	R	R	R	R	R
Gentamycin	10	S	R	S	R	R
Kanamycin	30	S	S	R	R	I
Norfloxacin	10	S	S	S	S	S
Streptomycin	10	I	R	R	R	R
Tetracycline	30	S	R	R	R	S

Antibiotic sensitivity is considered according to the zone diameter interpretative standards of CLSI [26]. R: resistant; I: intermediate, and S: sensitive.

**Table 2:** Antimicrobial activity of NTME and its solvent fractions against five strains of *Salmonella typhimurium*.

	KCTC 2515	ST171	ST482	ST688	ST21A
MIC (*μ*g/mL)					
NOR^a^	0.063	0.063	0.125	0.063	0.125
NTME	6250	6250	6250	6250	6250
DFNTE	>2500	>2500	>2500	>2500	>2500
EFNTE	781	781	>781	781	781
BFNTE	6250	6250	6250	6250	6250
MBC (*μ*g/mL)					
NOR^a^	1	1	1	1	1
NTME	12500	12500	25000	12500	12500
DFNTE	>2500	>2500	>2500	>2500	>2500
EFNTE	1562	1562	1562	1562	1562
BFNTE	25000	25000	25000	25000	25000

NOR: norfloxacin, NTME: *N. tetragona* 50% methanol extract, EFNTE: ethyl acetate fraction of *N. tetragona* 50% methanol extract, DFNTE: dichloromethane fraction of *N. tetragona* 50% methanol extract, BFNTE: butanol fraction of *N. tetragona* 50% methanol extract. ^a^Positive control.

**Table 3:** Fractional inhibitory concentration (FIC) and FIC index (FICI) of combination between tylosin and EFNTE.

Tylosin		EFNTE		FICI	Interpretation
Con^c^ (*μ*g/mL)	FIC of drug A	Con^c^ (*μ*g/mL)	FIC of drug B	(FIC of A + FIC of B)	
1024	MIC of A	0	MIC of A	MIC of A	MIC of A
1024	1	6	0.0077	1.008	IND
1024	1	12	0.0154	1.015	IND
1024	1	24	0.0307	1.031	IND
512	0.5	49	0.063	0.563	ADI
256	0.25	98	0.125	0.375	SYN
256	0.25	195	0.25	0.499	SYN
128	0.125	391	0.5	0.626	ADI
64	0.0625	391	0.5	0.563	ADI
0	MIC of B	781	MIC of B	MIC of B	MIC of B

EFNTE: ethyl acetate fraction of *N. tetragona* 50% methanol extract; Con^c^: concentration; drug A: tylosin; drug B: EFNTE; SYN: synergistic effect; ADI: additive effect; IND: indifferent effect; MIC: minimum inhibitory concentration. The FICI was interpreted as follows: synergistic effect (0 < FICI ≤ 0.5), additive effect (0.5 < FICI ≤ 1), and indifferent effect (1 < FICI ≤ 4).

**Table 4:** Fractional inhibitory concentration (FIC) and FIC index (FICI) of combination between streptomycin and EFNTE.

Streptomycin		EFNTE		FICI	Interpretation
Con^c^ (*μ*g/mL)	FIC of drug A	Con^c^ (*μ*g/mL)	FIC of drug B	(FIC of A + FIC of B)	
32	MIC of A	0	MIC of A	MIC of A	MIC of A
32	1	6	0.0077	1.008	IND
16	0.5	12	0.0154	0.515	ADI
16	0.5	24	0.0307	0.531	ADI
16	0.5	49	0.063	0.563	ADI
16	0.5	98	0.125	0.625	ADI
16	0.5	195	0.25	0.75	ADI
16	0.5	391	0.5	1	IND
8	0.25	781	1	1.25	IND
4	0.125	781	1	1.125	IND
2	0.0625	781	1	1.063	IND
0	MIC of B	781	MIC of B	MIC of B	MIC of B

EFNTE: ethyl acetate fraction of *N. tetragona* 50% methanol extract; Con^c^: concentration; drug A: streptomycin; drug B: EFNTE; ADI: additive effect; IND: indifferent effect; MIC: minimum inhibitory concentration. The FICI was interpreted as follows: synergistic effect (0 < FICI ≤ 0.5), additive effect (0.5 < FICI ≤ 1), and indifferent effect (1 < FICI ≤ 4).

**Table 5:** Quorum sensing inhibition activity (as pigment inhibition zone diameters) of *Nymphaea tetragona* 50% methanol extract and solvent fractions of the crude extract against *C. violaceum*.

Solvent fraction/antibiotic	Concentration	Pigment inhibition diameter (mm)
	(*μ*g/disk)	Mean ± SD
Tetracycline	10	16.33 ± 0.58*
Furanone	100	16.67 ± 0.58^a^
Normal Saline	—	ND
NTME	600	13.67 ± 1.15^c^
DFNTE	600	13.33 ± 0.58^c^
EFNTE	600	14.67 ± 1.15^b^
BFNTE	600	14.33 ± 1.53^b^

NTME: *N. tetragona* 50% methanol extract, DFNTE: dichloromethane fraction of *N. tetragona* 50% methanol extract, EFNTE: ethyl acetate fraction of *N. tetragona* 50% methanol extract, BFNTE: butanol fraction of *N. tetragona* 50% methanol extract. *Diameter of clear zone. ND: no activity detected. Data shown represent the mean ± SD of three replicates. Different alphabets indicate significant difference.

**Table 6:** Major compound list of three solvent fractions according to their contribution in respective solvent and compounds were listed followed by their elution order including reported activity.

RT	% area	Compound	Activity	Reference
Dichloromethane fraction of *Nymphaea tetragona* 50% methanol extract				
35.46	3.18	Heneicosan	—	
37.12	5.44	Tetracosan	—	
38.71	7.9	Pentacosan	—	
40.24	11.18	Hexacosan	Antimicrobial effects	[35]
41.71	10.57	Heptacosan	Antifungal activity	[35]
43.14	8.19	Octacosan	Antimicrobial effects	[35]

Ethyl acetate fraction of *Nymphaea tetragona* 50% methanol extract				
14.37	5.9	6,8-Dimethylbenzocyclooctene	—	
15.79	20.61	Pyrogallol	Antibacterial, QS inhibition, and potent tyrosinase inhibitor.	[36–39]
16.79	1.31	1,4-Cyclohexanedicarboxylic acid, 2,5-dioxo-diethyl ester	Anticolon cancer.	[40]
26.90	1.39	1-[7′-Methylbenzofuran-2′-carbonyl]-3-ethylazulene	Derivatives have antibacterial, antifungal, anti-inflammatory, analgesic, antidepressant, anticonvulsant, antitumor, anti-HIV, antidiabetic, antitubercular activity.	[41, 42]
27.65	70.44	Methyl gallate	Antimicrobial	[43]

Butanol fraction of *Nymphaea tetragona* 50% methanol extract				
12.86	57.61	2-Hydrazinoquinoline	—	
15.8	22.09	Pyrogallol	Antibacterial, QS inhibition, and potent tyrosinase inhibitor.	[36–39]
27.39	12.77	Methyl gallate	Antibacterial	[43, 44]

## Abbreviations

NTME: * Nymphaea tetragona* 50% methanol extract
DFNTE: Dichloromethane fraction of* Nymphaea tetragona* 50% methanol extract
EFNTE: Ethyl acetate fraction of* Nymphaea tetragona* 50% methanol extract
BFNTE: Butanol fraction of* Nymphaea tetragona* 50% methanol extract
GC-MS: Gas chromatography-mass spectrometry.
